# Disentangling Somatosensory Evoked Potentials of the Fingers: Limitations and Clinical Potential

**DOI:** 10.1007/s10548-017-0617-4

**Published:** 2018-01-20

**Authors:** Konstantina Kalogianni, Andreas Daffertshofer, Frans C. T. van der Helm, Alfred C. Schouten, Jan C. de Munck, on behalf of the 4DEEG consortium, Gert Kwakkel, Carel G. M. Meskers, Erwin E. H. van Wegen, Aukje S. Andringa, Dirk Hoevenaars, Caroline Winters, Sarah Zandvliet, Teodoro Solis-Escalante, Yuan-Yang, Martijn P. Vlaar, Lena Filatova, Julius P. A. Dewald, Jun Yao

**Affiliations:** 1 Department of Biomechanical Engineering, Delft University of Technology, Mekelweg 2, 2628 CD Delft, The Netherlands; 2 Department of Radiology and Nuclear Medicine, VU University Medical Center, Amsterdam, The Netherlands; 3 Amsterdam Movement Sciences & Institute for Brain and Behavior Amsterdam, Faculty of Behavioural and Movement Sciences, Vrije Universiteit Amsterdam, Amsterdam, The Netherlands; 4 MIRA Institute for Biomedical Technology and Technical Medicine, University of Twente, Enschede, The Netherlands

**Keywords:** Somatosensory evoked potentials, EEG, Reproducibility, Representation of fingers, Clinical biomarkers

## Abstract

In searching for clinical biomarkers of the somatosensory function, we studied reproducibility of somatosensory potentials (SEP) evoked by finger stimulation in healthy subjects. SEPs induced by electrical stimulation and especially after median nerve stimulation is a method widely used in the literature. It is unclear, however, if the EEG recordings after finger stimulation are reproducible within the same subject. We tested in five healthy subjects the consistency and reproducibility of responses through bootstrapping as well as test–retest recordings. We further evaluated the possibility to discriminate activity of different fingers both at electrode and at source level. The lack of consistency and reproducibility suggest responses to finger stimulation to be unreliable, even with reasonably high signal-to-noise ratio and adequate number of trials. At sources level, somatotopic arrangement of the fingers representation was only found in one of the subjects. Although finding distinct locations of the different fingers activation was possible, our protocol did not allow for non-overlapping dipole representations of the fingers. We conclude that despite its theoretical advantages, we cannot recommend the use of somatosensory potentials evoked by finger stimulation to extract clinical biomarkers.

## Introduction

Somatosensory impairment is highly associated with stroke severity (Connel et al. 2008; Meyer et al. 2016). More specifically, regaining individual finger function is considered a good predictor for recovery of upper limb function post stroke (Nijland et al. 2010). Whether this marks recovery of efferent or afferent connections from motor or somatosensory areas is a matter of dispute. In the current study, we focused on the latter and asked whether electric stimulation of the fingers may yield reliable responses in sensory areas as assessed by electro-encephalography (EEG). We tested for the candidate capacity of responses to finger stimulation as a clinical biomarker in general and more specifically for stroke recovery.

Somatosensory evoked potentials and fields (SEPs and SEFs, respectively) induced by electrical or mechanical stimulation on the median nerve is a well-established approach to investigate the electrophysiological phenomena linked to impaired somatosensation occurring, for example, while recovering from a stroke (Keren et al. 1993; Péréon et al. 1995; Timmerhuis et al. 1996; Rossini et al. 1998a, 2001; Wikström et al. 1999, 2000; Hari and Forss 1999; Feys et al. 2000; Tzvetanov and Rousseff 2003; Huang et al. 2004; Oliviero et al. 2004; Tecchio et al. 2007a, b, 2006; Al-Rawi et al. 2009). Early components of median nerve SEPs may indicate whether afferent connections arrive at the contralateral primary somatosensory cortex (S1). By stimulating directly at the median nerve, however, both cutaneous muscle and joint afferents are stimulated and potentially efferent fibers intervening (muscles) (Dawson 1956; Mauguiere 1999; Kuiken et al. 2007). Dependent on the intensity of the stimulus, finger stimulation will excite primarily Αβ fibers (Dowman 1997), followed by Aδ, followed by C fibers (McAllister et al. 1995; Kandel et al. 2000) while median nerve stimulation includes additionally sensory and motor fibers of larger diameter and partially the ulnar nerve. In view of our interest on hand representation, we hence focused on activity induced by stimulation of the digits, as assuming this to elicit responses at a more specified area at the somatosensory cortex.

Somatotopic arrangement and discrete representation of the fingers in the human cortex is well studied in the literature; Penfield and Boldrey (1937) already showed a systematic arrangement of representation of the human fingers on the cortex using intraoperative electrocorticography (ECoG), which was later confirmed by Penfield and Rasmussen (Penfield and Rasmussen 1950). Studies using local field potential recordings in animals revealed the refined spatial representation differentiating the input from different fingers (Kaas 1983), in particular in area 3b of SI. Over the last decade or so, high-resolution fMRI studies confirmed the somatotopic arrangement in area BA 3b reporting inter-digit distances that varied from 3.7 to 15.5 mm (van Westen et al. 2004; Martuzzi et al. 2014; Pfannmöller et al. 2015). M/EEG studies concentrated mainly on the representation of 1st and 5th digit. Using EEG, Baumgartner et al. (1993) revealed a distance of 12.5 mm between representations of thumb and little finger. Buchner et al. (1994) reported a somatotopic arrangement for two of three subjects tested. Barbati et al. (2006) found statistically significantly different representations for 1st and 5th finger with MEG that Houzé et al. (2011) confirmed with EEG, and the differences between ulnar and median nerve representation were found more significant. In the MEG studies of Rossini et al. (2001) and Rossini et al. (1998a) discrimination of the 1st and 5th digit was shown possible both for healthy controls and stroke patients, where enlargement of the hand area occurred.

Although the somatotopy of evoked responses has been addressed in various studies, it is still unclear how reliable and reproducible those responses are within and across subjects, both at sensor and at source level. In particular, it is unknown whether somatotopy of all the fingers can be demonstrated with EEG in individual subjects and in a reproducible way. The heterogeneity in stimulus protocols and the lack of datasets where all fingers are stimulated render the findings described in the literature difficult to judge. Using a pneumatic stimulation protocol, Schaefer et al. (2002) sketched test-rest reliability and reported a mean Euclidean distance of 7.42 mm between sources activations revealed by EEG measurements separated 1 month in time. However, this study did not address the possibility to discriminate non-overlapping representation of all the fingers in the somatosensory cortex with the use of EEG. We consider such a discrimination crucial when interested in using finger SEPs as potential biomarker.

In the present study, we assessed the test–retest variability of the responses. We also tested for the number of trials needed to obtain robust topographies and examined the possibility of discriminating different fingers at the cortex. Ultimate goal was to examine the possibility of using EEG and SEP on the finger as a subject-specific biomarker.

## Materials and Methods

### Participants

Five healthy volunteers participated in the study (1 left handed; mean ± SD age: 34 ± 12; 3 male 39 ± 13 years; and 2 female: 26 ± 3 years). Measurements were scheduled in four consecutive working days. The subjects had no previous or current neurological/motor deficits. They provided written informed consent, prior to the start of the experiment. The experimental protocol was in compliance with the declaration of Helsinki and approved by the institutional ethics committee of the Faculty of Human Movement Science, Vrije Universiteit Amsterdam, The Netherlands (ECB 2014-72). We note here, that this is an exploratory study and therefore we included a rather small sample size of five healthy volunteers. In order to suggest SEP induced by electrical stimulation of the finger as a relevant patient-specific biomarker, reproducible SEP responses and discrimination of finger representations should be possible in all five healthy participants tested.

### Experiment

#### Experimental Setup

**Fig. 1:**
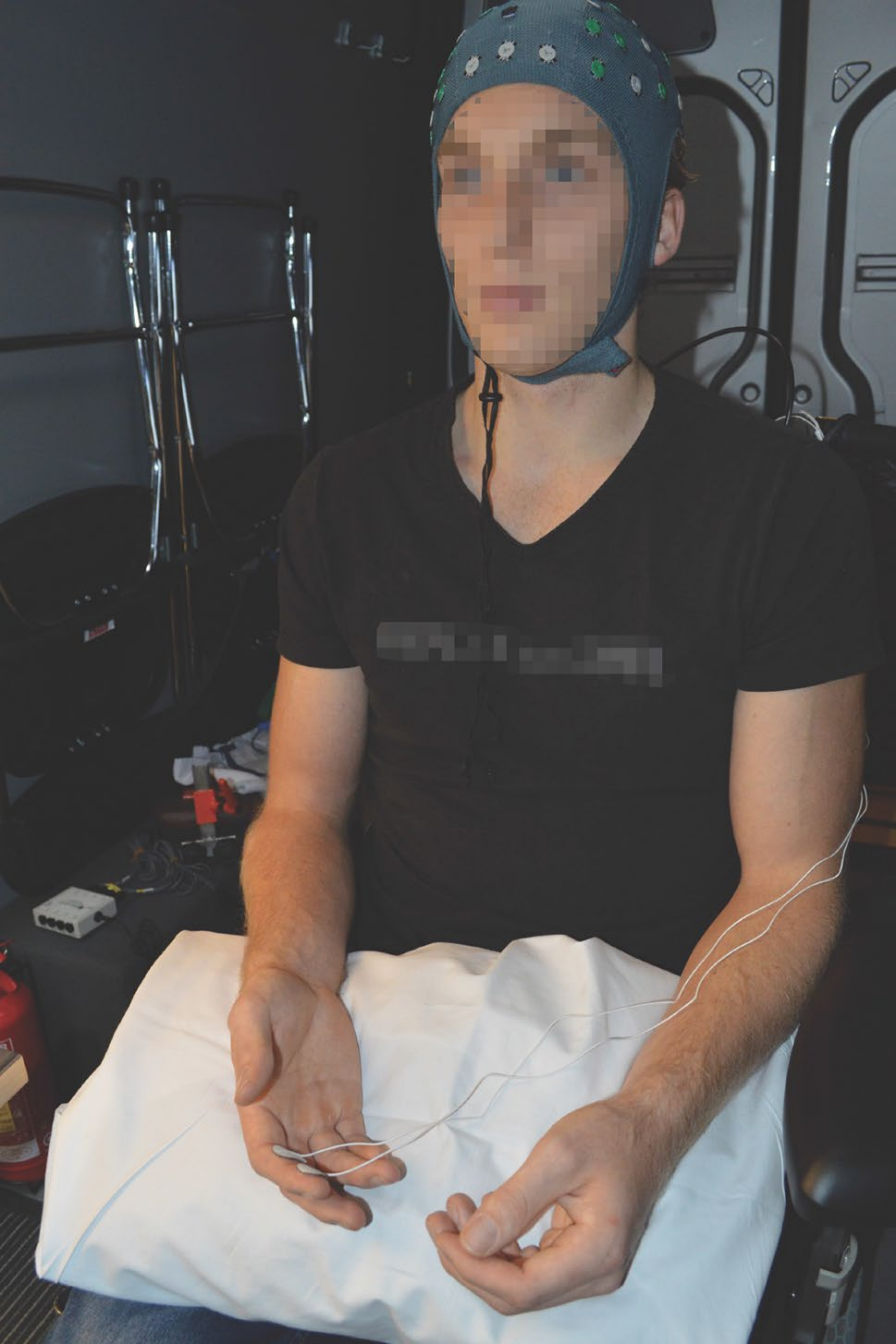
Experimental setup. One participant is seated on a wheelchair inside the experimental van with his dominant hand on a supine position, placed on a pillow. Here, the electrodes are attached on his index finger

During the experiment, participants were sitting comfortably with their dominant hand and forearm positioned on their lap with the fingers on top (supine position). Between forearm and lap a pillow was placed to secure a stable position and comfort, as depicted in Fig. 1. The experiment was performed within a NEN1010 approved measurement van, which was equipped with high-density EEG located at the VU University Medical Center (Amsterdam, The Netherlands).

Data were recorded with a 64-channel EEG system (TMSi, Netherlands) with ground electrode placed at the left mastoid and referenced to the common average during recording. Sampling rate was 1024 Hz and apart from anti-aliasing filters no other filters were applied online. Positions of the EEG electrodes for every subject were measured with the ANT Neuro Xensor system (ANT Neuro, Enschede, Netherlands).

Electric stimuli were delivered to the fingers with a bipolar battery-powered electrical stimulator by Micromed (Brain quick) in order to record the somatosensory evoked potentials (SEPs) with EEG. Two electrodes (bipolar stimulation) were applied to all the fingers. The anodal stimulation was placed at the most distal phalange of the finger and the cathode at the second most distal phalange of the finger with an inter-electrode distance of approximately 1 cm. A monophasic anodic rectangular electrical pulse of 400 μs width and a stimulation intensity of two times the sensation threshold was chosen. The sensation threshold was defined as the level at which the subject was able to sense half of the 10 pulses given. To define the aforementioned parameters, we reviewed the literature and we tested for effects of the electrical pulse’s width and intensity on the amplitude and reproducibility of the SEP responses on one participant prior to conducting the experiment; more information can be found in the Appendix. The chosen stimulation did not cause any inconvenience to the participants.

#### Experimental Protocol

Participants were instructed to relax neck, shoulder, and face muscles, to blink normally, to avoid talking or swallowing, and to fixate their gaze at a cross at a computer screen about 60 cm in front of them. All fingers of the dominant hand were electrically stimulated in random order.

The five conditions of the experiment (corresponding to the five different fingers) were repeated in two blocks, with approximately 10–15 min difference between the two blocks. Each condition consisted of 500 trials (repetitions of stimuli). Different (random) orders of stimulation were used in both blocks of the experiment. The inter-stimulus interval (ISI) was chosen from 250 to 330 ms (varied randomly), as the later responses at the somatosensory area are expected up to 110 ms (Hämäläinen et al. 1990) and ISIs larger than 150 ms do not affect the deflections of P35 and P60 (Wikström et al. 1996). Between every finger’s stimulation there was a break of at least 1 min but not longer than 3 min according to participant’s needs.

### Pre-Processing of the Data

The data were pre-processed offline using Matlab (R2013b; The Mathworks, Natick, MA) with the Fieldtrip (Oostenveld et al. 2011) and EEGlab toolboxes (Delorme and Makeig 2004). After linear interpolation of the stimulation artefact (lasting for approximately 6 ms after stimulus onset), data were band-pass filtered between 1 and 250 Hz using a bi-directional 4th order Butterworth filter. The data were segmented in 250 ms stimulus-locked epochs including a 50 ms pre-stimulus interval. Noisy epochs and channels were identified visually and discarded. Artifact-free data consisted of about 780 trials (78% of the total number of trials) and about 50 channels (78% of the total number of channels). After re-referencing to the average of the remaining channels, the SEPs were computed for each dataset.

### Signal-to-Noise Ratio (SNR)

The SNR per channel and sample was calculated via:

1 $$SNR(c)=\sqrt {\frac{{\sum\limits_{{t=1}}^{T} {\mathop {^{{\overline {{x(c,t)}} }}}\nolimits^{2} } }}{{\sum\limits_{{t=1}}^{T} {{{\operatorname{var} }_n}(x(n,c,t))} }} \cdot N} $$

where *x* is a 3-way data matrix consisting of *n* trials, *c* channels, *t* time samples. *N* is the total number of trials. The power of the averaged signal $\overline {x}$ over trials was calculated by taking the sum of squares of all samples T over a specific time window (20–120 ms after stimulus onset) and then divided by the sum of the variance over trials ${\operatorname{var} _n}$. This ratio was multiplied by the total number of trials and the square root of this portion served as measure of SNR.

The SNR was averaged over all the channels resulting in one SNR value for every subject and every finger.

### Dipole Fitting

A dipole fit for all the subjects and conditions of the experiment was computed with a current dipole algorithm as described in De Munck et al. (2001). The current dipole algorithm splits the inverse problem into the linear and non-linear part and then performs a global search based on a fixed grid with mesh size of 1 cm, followed by a full non-linear search. A concentric three-sphere head model was determined by fitting a sphere on the subject-specific electrode positions. For the inner and outer radii of the skull fixed ratios with respect to the fitted head radius were used. The dipole fit was applied to the P50 peak that was identified for every subject and for every condition after computing the global field power (μV^2^) at the BIAP software (http://www.demunck.info/software/index.html). By this, individual differences were accounted for.

### Statistics

#### Spatio-Temporal Reproducibility

The spatio-temporal reproducibility of the SEPs was quantified as the correlation coefficient *r* between two averages *A*_*ct*_ and *B*_*ct*_, where *A*_*ct*_ and *B*_*ct*_ represent two averaged responses and *c* refers to channel and *t* to sample. Here *t* is varied over a specific time window corresponding to the signal’s peak. The correlation coefficient was calculated similar to Goszczynska et al. (2014) and it is as follows:

2 $${\text{r}}=\frac{{\sum\limits_{c}^{{}} {\sum\limits_{t}^{{}} {(A(c,t) - \overline {A} )} } (B(c,t) - \overline {B} )}}{{\sqrt {\left( {\sum\limits_{c}^{{}} {\sum\limits_{t}^{{}} {{{(A(c,t) - \overline {A} )}^2}} } } \right)\left( {\sum\limits_{c}^{{}} {\sum\limits_{t}^{{}} {{{(B(c,t) - \overline {B} )}^2}} } } \right)} }}$$

Here $\overline {A}$ and $\overline {B}$ are determined by averaging the SEPs over channels and time samples. With the use of *r* the whole spatiotemporal pattern of the responses is taken into account. *r* is used to quantify the similarity of the SEPs belonging to the same experimental condition but consisting of different subset of trials. The values of *r* range between − 1 and 1, where 1 defines full correlation and − 1 full negative correlation. As proposed by (Goszczynska et al. 2014) a value of *r* = 0.9 identifies similar EEG spatiotemporal patterns.

#### Bootstrapping

We used a resampling bootstrapping technique in order to set confident regions of the responses at electrodes and sources level (Darvas et al. 2005). By randomly drawing a specific number of trials out of the total number of trials (780 ± 50) a bootstrap of the average response was constructed for a specific number of trials. We took 100 random subsets of a certain percentage of the trials and for each subset we computed the averaged SEP response. The percentages of trials were varied in steps of 10% from 10 to 90%, in order to determine the requested number of trials for a reproducible response.

For every bootstrapped-based average, a dipole was computed, resulting in 100 dipoles for every condition. Each dipole is represented in 3D space in (*x, y, z*)-coordinates. Nasion, left pre-auricular point (LPA), and right pre-auricular point (RPA) coordinates were used, with + *x* pointing to the nose, + *y* pointing to the left ear and + *z* pointing to the top of the head. To quantify the findings, we computed for every condition the mean location *m* of the cloud of 100 dipoles and the position covariance matrix $C$. Parameter variations of the reconstructed dipole locations are illustrated with an ellipse centered at the $m$ dipole position. The axes of the ellipsoids are oriented according to the principal axes of variation of each cloud of dipoles. The principal axes are computed as the eigenvectors of the $C$. For specifying the overlap of the ellipsoids corresponding to the bootstrapped-based representation of the fingers i and j we computed the pre-whitened distance between the cluster’s centroids i and j. Assuming that the ellipsoids have independent Gaussian distributions with covariances ${C_i}$ and ${C_j}$, then the distribution of their difference is Gaussian, with mean ${m_i} - {m_j}$ and covariance ${C_i}+{C_j}$. If ${W_{ij}}$ is a pre-whitening matrix, such that ${W_{ij}}W_{{ij}}^{T}={\left( {{C_i}+{C_j}} \right)^{inv}}$, the possible separability of the two ellipsoids is defined by the length of the vector $z$

3 $$z={W_{ij}}\left( {{m_i} - {m_j}} \right)$$

The (dimensionless) measure of separability ${S_{ij}}$ of the two ellipsoids is defined as

4 $${S_{ij}}=\sqrt {\mathop z\nolimits_{x}^{2} +\mathop z\nolimits_{y}^{2} +\mathop z\nolimits_{z}^{2} } $$

When ${S_{ij}}$ is larger than 2 the ellipses corresponding to the dipoles’ representations are considered as not overlapping and when ${S_{ij}}$ is smaller than 2 the ellipses overlap.

## Results

### Global Field Power of the SEP Responses

**Fig. 2:**
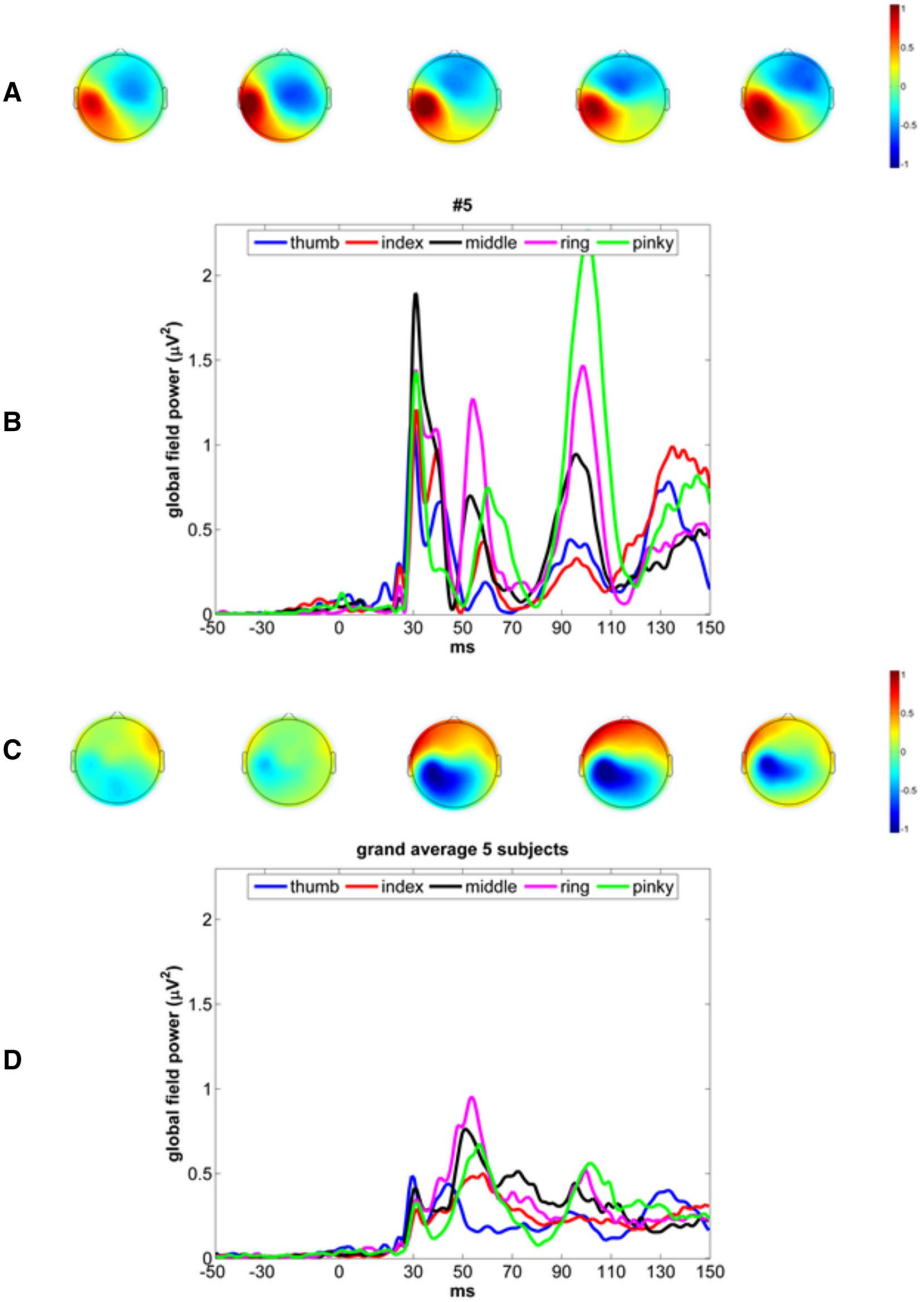
**a** Topographical distribution of the P30 peak. **b** GFPs of SEP responses after stimulation of the individual fingers of subject 5. Different colors represent different fingers. **c** topographical distribution of the P50 peak. **d** GFP of grand average of SEP responses of 5 subjects

The global field power (GFP) computed in μV^2^ (Brunet et al. 2011) for different fingers is plotted for participant 5 in Fig. 2. The GFP was used to identify peaks in the event-related signal (Michel and Murray 2012). Although the peaks varied between participants and fingers, typically three peaks could be observed in the SEP: one peak around 30 ms, one peak around 50 ms (P50 peak) and one peak around 100 ms. Since we were particularly interested in the early responses that correspond to S1 activation, a window of 25–65 ms after stimulus onset was selected for further analysis. The P50 was always present in the SEPs of all subjects in contrast to the P30. We thus selected P50 as the candidate peak for source localization.

### Signal-to-Noise Ratio

The SNR was determined as explained in “Statistics” for the time window 25–65 ms post-stimulus. It was found to be (SNR ± SD): 2.6 ± 0.8 for subject 1, 3.4 ± 0.5 for subject 2, 3.8 ± 0.5 for subject 3, 2.4 ± 0.6 for subject 4, and 3.3 ± 0.5 for subject 5. Here, standard deviations (SD) were computed over fingers. Accordingly, the SNR for the pre-stimulus window − 40 to 0 ms was found to be (SNR ± SD): 1.7 ± 0.5 for subject 1, 1 ± 0.1 for subject 2, 1 ± 0.1 for subject 3, 1.5 ± 0.3 for subject 4, and 1 ± 0.2 for subject 5.

### Reproducibility of SEP Responses

#### Test–Retest Reproducibility

**Fig. 3:**
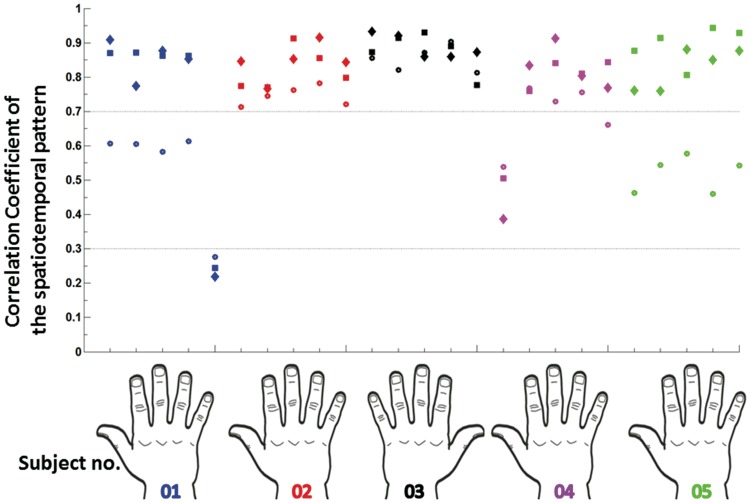
Reproducibility of the SEP. The spatiotemporal correlation coefficient was computed for the window of 25–65 ms after stimulus onset. The diamond markers represent the correlation between the 2 experimental repetitions of the same finger, the ‘o’ markers represent the correlation of one finger with all the others and the square markers refers to the correlation coefficient of 2 randomly selected subsets after stimulation of the same finger. Each subject is associated with a specific color and the correlation coefficients of every finger are plotted above the fingers of the hand representation. Subject 3 was left handed

The correlation coefficients for the two experimental runs over a time window 25–65 ms are shown in Fig. 3. We selected a window that most probably contains two peaks, because we want to test the reproducibility of the topographical distribution, generated after electrical stimulation. The mean correlation coefficient was 0.77 ± 0.14 and the mean correlation coefficients per finger were, thumb: 0.74 ± 0.20, index: 0.77 ± 0.06, middle: 0.86 ± 0.02, ring: 0.85 ± 0.05, pinky: 0.70 ± 0.30, where standard deviations indicate inter-subject variability. This indicates that the middle finger yielded the most reproducible response, with a similarity close to 0.9. To address the habituation effects that might occur at the second experimental run, we constructed two datasets containing randomly selected non-overlapping subsets of trials (50% of the trials in each dataset) and then the correlation coefficient was computed as shown in Fig. 3. Small differences were present in the correlation coefficient of the two runs, however they had a similar pattern.

The computation of the correlation coefficient between the SEP responses of all combinations of fingers shows that the spatiotemporal patterns of all the fingers did not differ significantly (mean correlation coefficient 0.67 ± 0.15) from the patterns of all the other fingers, as also supported from Wang et al. (2004) for index-pinky spatiotemporal correlation coefficient. In Fig. 3 this is indicated per subject with the “o” markers. This finding suggests that at least at sensor level there was no large distinct spatiotemporal pattern per finger.

#### Number of Trials Needed for Reproducible Responses

**Fig. 4:**
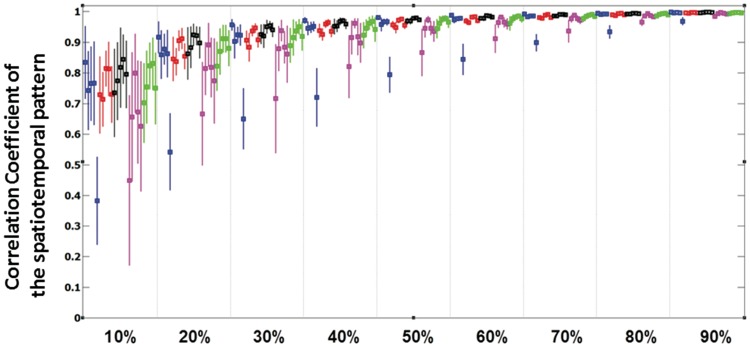
Correlation coefficient of X % trials averaged pattern and true averaged pattern (100% of trials). The mean and standard deviation of the correlation coefficient is shown, computed from 100 randomly selected time windows from 25 to 65 ms. Every percentage block shows means and standard deviations for all subjects and fingers. The same color code for the different fingers is used as in Fig. 3

A bootstrapping-resampling technique served to assess the effect of the number of trials on the reconstruction of the spatial–temporal responses patterns determined from the total number of trials. The quality of this reconstruction was quantified by computing the correlation coefficient between the SEP response computed with the total number of trials and a subset of a certain percentage of the trials. The mean and standard deviations of these correlations were computed over 100 random subsets, and displayed as a function of the percentage of trials in Fig. 4. Computations were conducted separately for all five fingers and five subjects. One observes that after including in the dataset 30% of the total number of trials it was possible to reproduce the original dataset by 85% (except two outliers). By including 50% of the trials (390 trials) a correlation coefficient of 95% was achieved on average. Specifically, after including 390 trials in the SEP response, the topographical distribution showed similarity to the response after including all trials and was: 0.93 for the thumb, 0.96 for the index, 0.97 for the thumb 0.94 for the ring and 0.96 for the pinky, as shown in Table 1. Those values exceeded the threshold 0.9 as indicated by (Goszczynska et al. 2014) and therefore the responses after including 50% of the trials (390) and 100% of the trials showed similarity.

**Table 1:** Percentage of trials needed for a reproducible response

	%30 of the total number of trials					%50 of the total number of trials				
	#1	#2	#3	#4	#5	#1	#2	#3	#4	#5
Thumb	0.96 ± 0.02	0.89 ± 0.03	0.92 ± 0.04	0.72 ± 0.16	0.88 ± 0.05	0.98 ± 0.01	0.95 ± 0.01	0.96 ± 0.01	0.87 ± 0.07	0.94 ± 0.03
Index	0.90 ± 0.05	0.88 ± 0.04	0.92 ± 0.04	0.88 ± 0.06	0.90 ± 0.06	0.96 ± 0.02	0.95 ± 0.02	0.97 ± 0.01	0.95 ± 0.03	0.96 ± 0.02
Middle	0.92 ± 0.02	0.92 ± 0.02	0.95 ± 0.02	0.94 ± 0.03	0.93 ± 0.03	0.97 ± 0.01	0.97 ± 0.01	0.98 ± 0.01	0.97 ± 0.01	0.97 ± 0.01
Ring	0.92 ± 0.03	0.94 ± 0.02	0.95 ± 0.02	0.89 ± 0.05	0.94 ± 0.03	0.97 ± 0.01	0.97 ± 0.01	0.98 ± 0.01	0.95 ± 0.02	0.97 ± 0.01
Pinky	0.67 ± 0.09	0.90 ± 0.03	0.94 ± 0.02	0.86 ± 0.08	0.92 ± 0.04	0.81 ± 0.05	0.95 ± 0.01	0.97 ± 0.01	0.94 ± 0.03	0.96 ± 0.02

Mean and standard deviation (mean ± std) of the spatio-temporal correlation coefficient of the averaged response after including 30 and 50% of the total number of trials and of the averaged response after including the total number of trials

### Representation of Different Fingers on the Sources Level

#### Location of the Centroids of the Bootstrapped-Based Dipoles

**Fig. 5:**
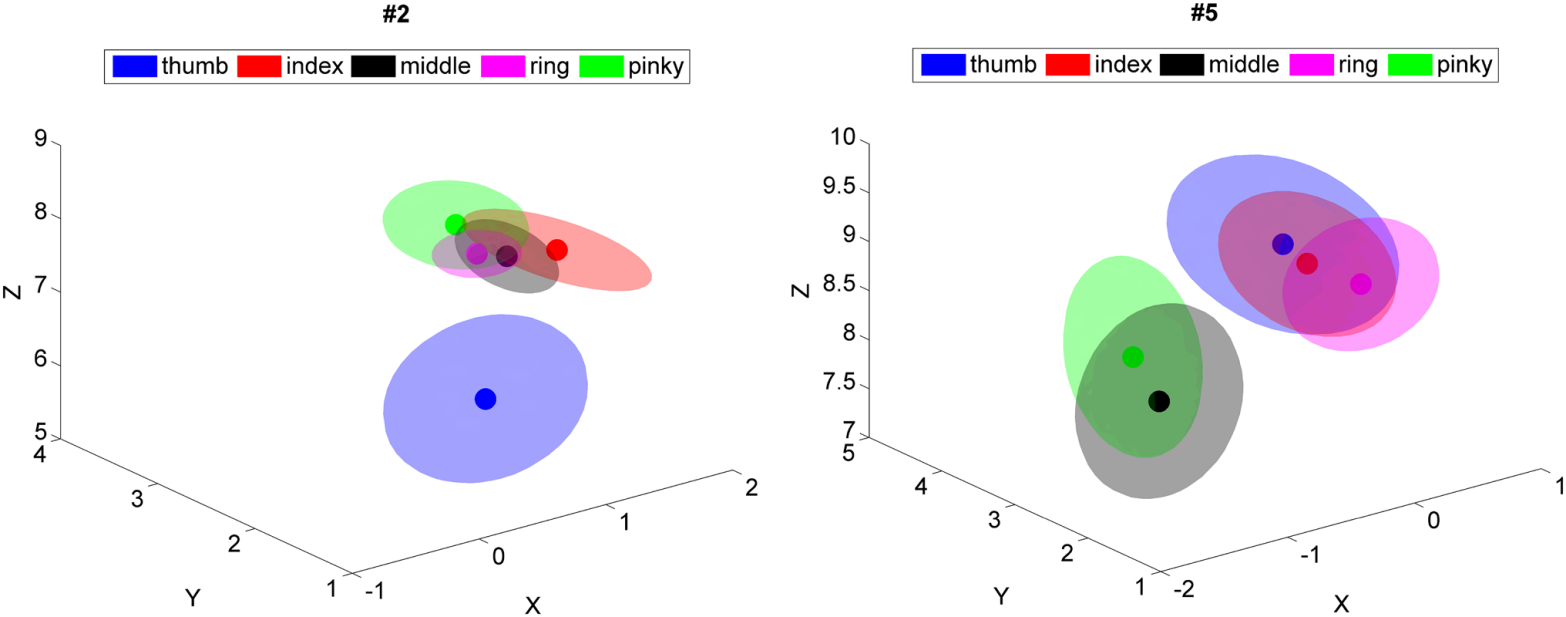
The mean dipole locations of the P50 components, estimated over 100 random subsets of trials are plotted for different fingers with filled dot. The ellipses around them represent the radius of confidence. Blue: thumb, red: index finger, black: middle finger, purple: ring finger and green: pinky. The half axes of the ellipsoids are two times the standard deviation of the spatial variation of the 100 bootstrapped based locations. The orientation of the ellipsoids is given by the eigenvectors of the covariance matrix of the dipole locations of each cloud of points. Dipole coordinates are represented in Nasion-Ear coordinates, in cm. On the left results of subject 2 are shown and on the right those of subject 5

In Fig. 5 the mean location per finger over the 100 permutations is shown as the cluster of the centroid (with a bold dot) and around this an ellipsoid is plotted with the axes as defined in “Bootstrapping”. For the two subjects whose data are displayed in Fig. 5 (subject 2 and 5) we observed no systematic order of the representation of the fingers while the estimated representations of fingers were strongly overlapping. In order to have a proper somatotopic arrangement, the values of *y*-coordinates should have had an ascending order from thumb to pinky. This arrangement could only be confirmed to some extent for subject 2 (see Table 2), although the radii of the representation ellipsoids (while being the smallest over subjects) did not allow for a complete separation of the representation of the fingers. The other four subjects did not show such somatotopic arrangement.

**Table 2:** Mean and confidence interval of dipoles’ location

	Finger	X	Y	z	Radius
Subject 1	Thumb	4.16	1.51	5.32	1.67
	Index	3.90	2.07	2.98	2.54
	Middle	4.4	1.01	3.42	2.00
	Ring	6.07	− 0.33	1.58	2.39
	Pinkie	2.89	4.98	3.18	4.18
Subject 2	Thumb	0.60	1.71	6.19	0.83
	Index	1.44	2.07	7.61	0.54
	Middle	1.14	2.20	7.58	0.40
	Ring	0.89	2.18	7.74	0.32
	Pinkie	1.07	2.63	7.78	0.51
Subject 3	Thumb	1.01	− 3.82	6.99	1.44
	Index	0.47	− 3.49	6.73	1.63
	Middle	− 0.10	− 3.72	7.76	1.01
	Ring	− 0.92	− 4.00	8.42	0.63
	Pinkie	0.38	− 2.49	6.52	1.71
Subject 4	Thumb	2.50	4.83	9.01	1.62
	Index	2.64	1.75	6.81	1.31
	Middle	3.69	3.11	6.55	1.30
	Ring	2.36	4.18	9.55	1.17
	Pinkie	2.25	3.59	8.32	1.35
Subject 5	Thumb	0.19	3.14	8.84	0.90
	Index	0.87	2.62	8.85	0.70
	Middle	− 1.06	2.65	7.85	0.84
	Ring	0.39	2.41	8.61	0.60
	Pinkie	− 0.83	3.42	7.95	0.83

Mean location and confidence radius of the dipoles estimated over 100 random subsets. Dipoles are computed for the P50 peak. Note that subject 3 is left-handed thus stimulated at left hand so location refers to right hemisphere. All values are in cm and nasion-ears coordinates are used

#### Overlap of the Fingers’ Representation

To illustrate the general overlap of the finger representations, we show in Table 3 the minimum and maximum separability measure over all subjects. For example, the separability index between middle and ring fingers amounts to 7.10 in the “best” subject, indicating that there was no substantial overlap between the bootstrapped-based representations for this subject. However, for the same combination of fingers, we found a subject in whom the separability was only 0.96 implying an almost complete overlap of the bootstrapped-based clusters. Since the figures represented in Table 3 are not caused by a single good or poor performing subject, we have to conclude that there was no pair of fingers for which good separability exists for all subjects. Specifically, the minimum part of the table shows there was no pair of fingers for which the separability was higher than 0.68 for all the subjects.

**Table 3:**
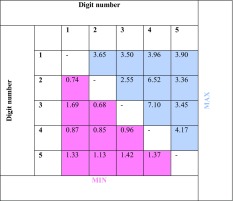
Maximum and minimum separability of fingers

The maximum and minimum separability over all subjects are shown for all finger combinations

## Discussion

In the present study, we evaluated the use of somatosensory evoked potentials induced by finger stimulation, as a potential biomarker tool for post-stroke recovery. We were especially interested in testing the robustness of such a biomarker as a lack of consistency would make it impossible to track longitudinal changes at a subject-specific level. The large overlap of the dipole representations for different fingers and the relatively low reproducibility of the test–retest design indicate the difficulty of separating the representations of different fingers as determined by EEG and electrical finger stimulation. Moreover, the somatotopic representation of the fingers could only be confirmed in one of the five subjects, whereas for the others it was not so consistent. We emphasize that these results were found in the ‘ideal’ condition in which an optimal stimulation protocol was used and reproducibility measure was not compromised by detaching and re-attaching the EEG cap.

Parts of these admittedly discouraging results are consistent with other studies. We found one or two peaks from 25 to 65 ms (Baumgartner et al. 1993; Buchner et al. 1994; Hari and Forss 1999; Wang et al. 2004; Schubert et al. 2008; Houzé et al. 2011; Bourguignon et al. 2013; Nierula et al. 2013) and a later peak around 100 ms (Hämäläinen et al. 1990). Note that the early peaks are often referred to as N20 and P27 in the literature in accordance with peaks found after median nerve stimulation, the latencies found after finger stimulation do not agree with 20 or 27 ms (Wang et al. 2004). In order to assess the test–retest repeatability of the responses and the number of trials needed for reproducible responses, we selected a window from 25 to 65 ms, since only a few studies referred to the later peaks. The early peak appears to correspond to activity in S1 (Forss et al. 1994). Even at the convenient setup of not removing the EEG cap, correlation coefficients were at mean (± SD) of 0.8 ± 0.16. The fairly low reproducibility was not affected by habituation effects in the test re-test design because the correlation coefficients of the random subset of trials are in the same range. Seeking to eliminate all parameters that may lead to irreproducible responses, we estimated the SNR with a similar way as Darvas et al. (2005) and the minimum number of trials needed in order to have repeatable spatiotemporal patterns. With an SNR of 2.9 [2.8 found in a MEG study, (Darvas et al. 2005)] we believe that our pre-processed signal was accurate. We found a minimum number of 230 trials to be needed for reproducing topographies that can be achieved with the total number of 780 trials. An indication about the variations at the brain responses is given by Darvas et al. (2005). We also observed a higher correlation coefficient for the middle finger and a lower correlation coefficient for the pinky, in accordance with small amplitudes, poor SNR and difficulty in source localization for the fifth digit reported in other studies (Baumgartner et al. 1993; Buchner et al. 1994; Houzé et al. 2011).

Although many studies applied electrical stimulation on the fingers to evoke SEPs or SEFs, as of yet there is no consensus on stimulus characteristics, nor explanation on the design of the chosen experimental protocol. To the best of our knowledge all studies used a monophasic anodic rectangular pulse of various pulse widths and intensities. MEG studies used a pulse width of 0.2 ms and an intensity of two times the sensory threshold or a pulse width of 1 ms and an intensity below the pain threshold (Kristeva-feige et al. 1995; Xiang et al. 1997; Darvas et al. 2005). Stimulation protocols among EEG studies were even more inconsistent. The width of electrical pulse found was 0.2, 0.3, 0.4 ms and the intensities varied as well as 1.5, 2, 3 times the sensory threshold, maximum comfortable level or below the pain threshold (Baumgartner et al. 1993; Buchner et al. 1994; Yao and Dewald 2005; Schubert et al. 2008; Houzé et al. 2011; Nierula et al. 2013). Inconsistency of stimulation protocols led us to test several stimulation parameters and their ability to produce reproducible responses (see Appendix). A more detailed test protocol with smaller steps between the pulse widths will be helpful in revealing the effect of the pulse width to the responses. However, this is not the purpose of the present study.

With a high SNR, a number of trials adequate for resulting in reproducible responses and a stimulation protocol optimal for finger stimulation, we further tested the representation of the fingers at the sources level. Although there is evidence for discrimination of the activation related to different fingers and mainly of thumb and pinky using EEG (Baumgartner et al. 1993; Buchner et al. 1994; Houzé et al. 2011; Nierula et al. 2013) or MEG (Buchner et al. 1994; Rossini et al. 1998a, 2001; Barbati et al. 2006) it was still unclear if this discrete representation of the fingers is prone to trial to trial variations or subject-specific differences. Darvas et al. (2005) addressed this topic with using MEG of one subject revealing the somatotopic arrangement of the fingers. However, they found for the S1 sources, a standard deviation of the 1000 locations of the bootstrapped dipoles between 3 and 5 mm revealing an overlap for some of the fingers representation. Our results show even higher standard deviation between 2 and 28 mm that may be explained by the poorer spatial resolution of EEG in comparison to MEG (Leahy et al. 1998) and the lower SNR of EEG concerning superficial sources (de Jongh et al. 2005; Goldenholz et al. 2009). As it can be seen in Table 2 we failed to pinpoint a pattern of somatotopic arrangement of the fingers for all the subjects, let alone a clear pattern of which fingers could be disentangled in the human cortex. The use of realistic head models in the dipole calculation might have resulted in more accurate dipole positions (Schaefer et al. 2002). In the current study, however, we were interested in relative locations of fingers in the brain and their reproducibility and not in exact 3D locations of every finger on the cortex.

EEG being an affordable and accessible technique along with single nerve recruitment by electrical stimulation of the finger can serve as a tool in the clinic, for example for monitoring stroke rehabilitation. However, the variability of the responses and the absence of a reproducible pattern of the finger somatotopy imply that the finger representation estimated with EEG is not a recommendable subject-specific monitoring tool for a longitudinal stroke study. MEG or fMRI along with electrical finger stimulation are modalities with higher spatial resolution (Rossini et al. 2001; Darvas et al. 2005), but patient’s ease and longitudinal monitoring will be at stake for patients having to travel to the hospital. If not only refined finger representation is of interest, median nerve stimulation could be used as an alternative. Electrical stimulation of the median nerve is a popular experimental choice when stroke assessment is of interest (Rossini et al. 1998a, b, 2001; Forss et al. 1999; Wikström et al. 2000, 1999; Hari and Forss 1999; Tecchio et al. 2001, 2007a; Huang et al. 2004). It induces several peaks including an early peak around 20 ms and one around 50 ms. Due to the fact that the median nerve stimulation activates both the sensory and motor areas, responses with larger amplitudes are observed when compared to the ones after electrical stimulation of the finger and with more prominent peaks. However, median nerve stimulation on stroke patients also showed limitations. For example in (Tecchio et al. 2007b) median nerve stimulation responses were identifiable at the affected hemisphere only in 56% of the patients. Yet, even when responses are identifiable we should expect an accuracy of 10 mm as indicated by Bourguignon et al. (2013) .A potential clinical use of the SEPs induced by finger stimulation could be the study of afferent pathways and brain areas recruited in S1 and S2 time locked to the appearance of early and late peaks.

## Conclusions

Distinct locations of fingers representation during electrical stimulation were found for all participants. However, in a sample of five healthy young participants we failed to find non-overlapping dipole confidence limits of different fingers for all the subjects using high-density EEG. Responses appeared variable within and across recording sessions and therefore SEP induced by electrical stimulation on the fingers and recorded with EEG as a tool for subject-specific clinical assessment (for example, longitudinal post-stroke assessment) should be used with great care.

## Figures and Tables

**Fig. 6:**
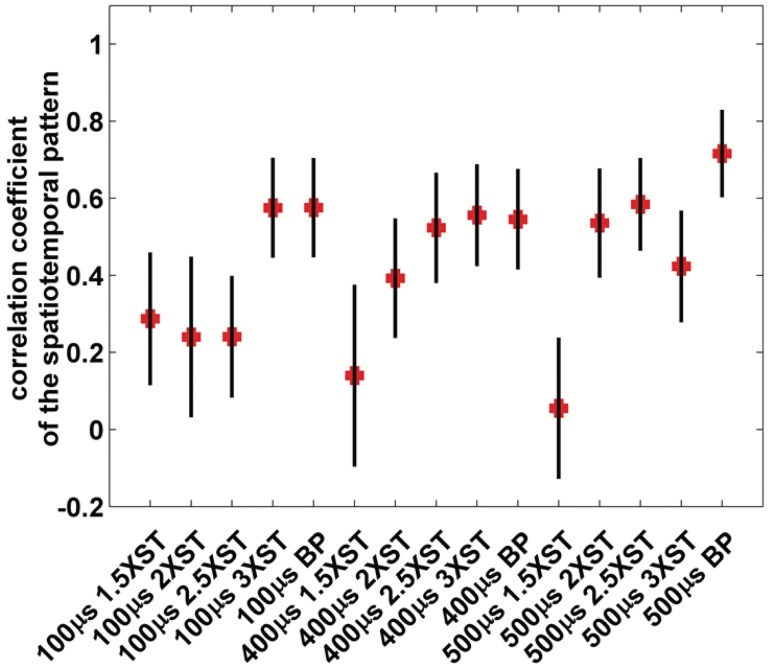
Correlation coefficient of the spatiotemporal patterns for the time window 25–65 ms post stimulus and for different stimulation protocols. Mean and standard deviation over the bootstrapped based correlations are shown

**Fig. 7:**
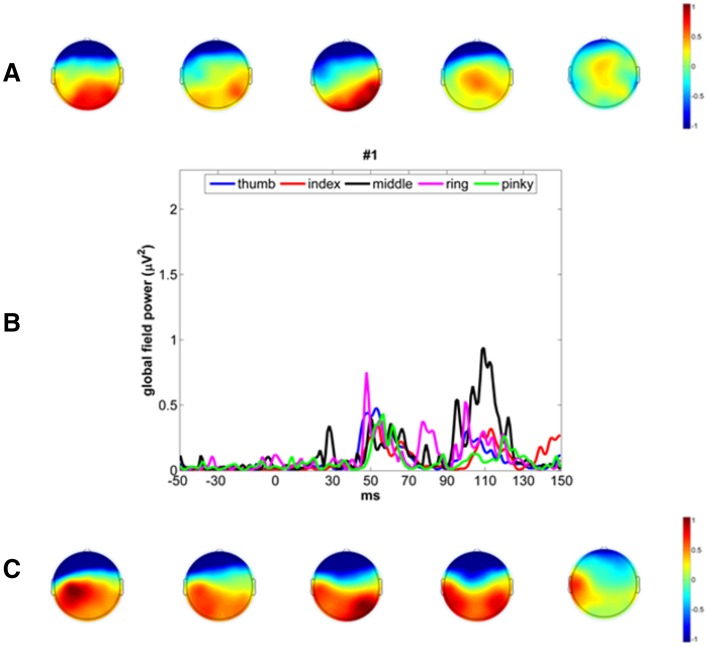
**a** Topographical distribution of the P30 peak. **b** GFPs of SEP responses after stimulation of the individual fingers of subject 1. Different colors represent different fingers. **c** topographical distribution of the P50 peak

**Fig. 8:**
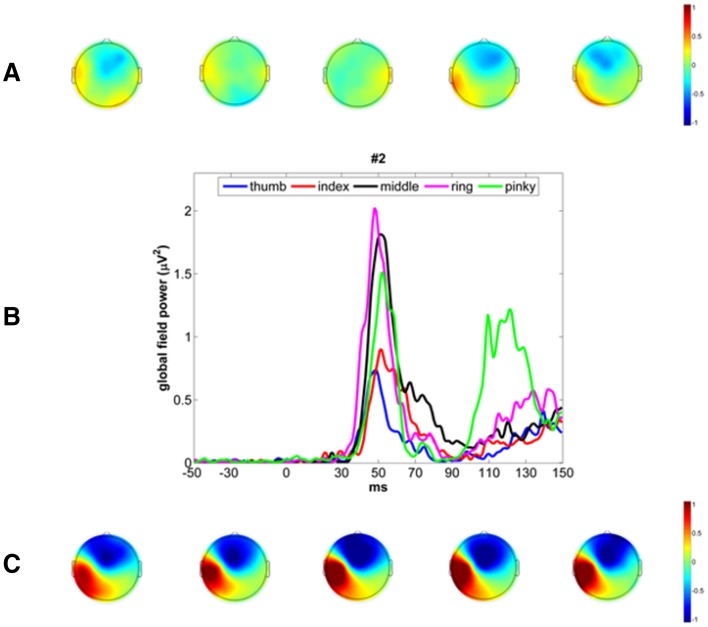
**a** Topographical distribution of the P30 peak. **b** GFPs of SEP responses after stimulation of the individual fingers of subject 2. Different colors represent different fingers. **c** topographical distribution of the P50 peak

**Fig. 9:**
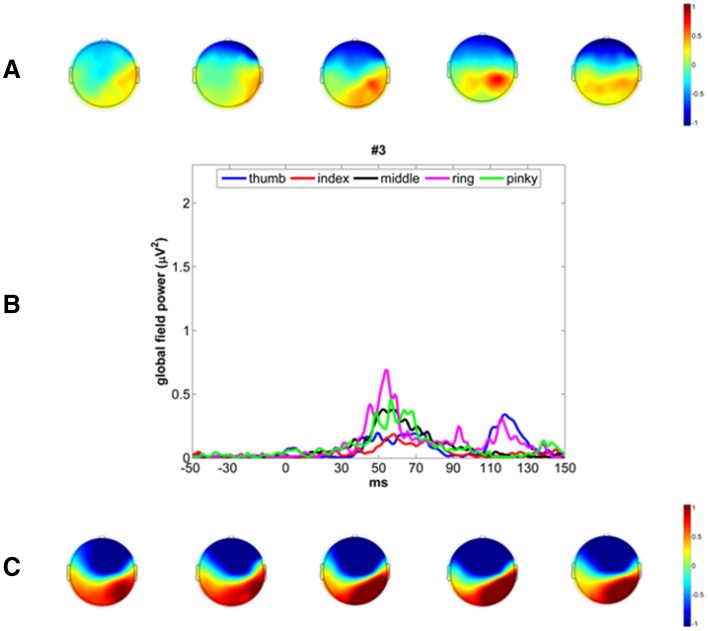
**a** Topographical distribution of the P30 peak. **b** GFPs of SEP responses after stimulation of the individual fingers of subject 3. Different colors represent different fingers. **c** topographical distribution of the P50 peak

**Fig. 10:**
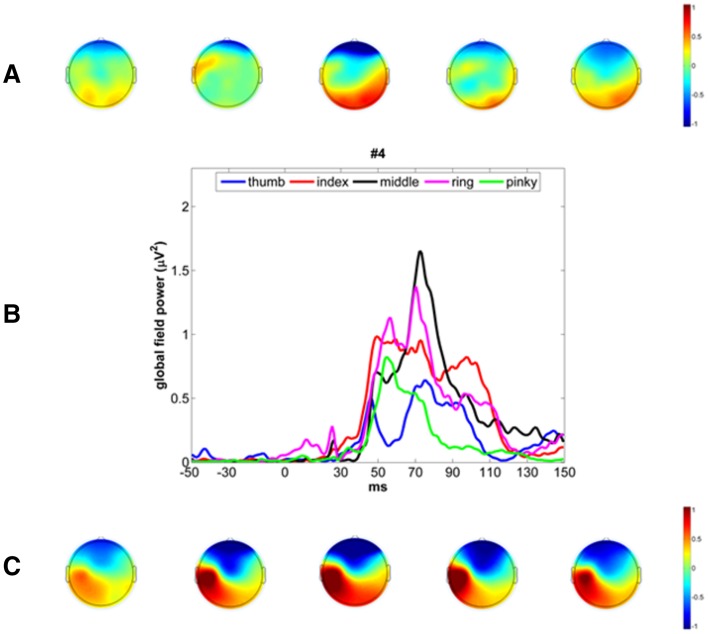
**a** Topographical distribution of the P30 peak. **b** GFPs of SEP responses after stimulation of the individual fingers of subject 4. Different colors represent different fingers. **c** topographical distribution of the P50 peak

## Appendix

### Definition of the Electrical Pulse

Prior to conducting the experiment, we tested for effects of the electrical pulse’s width and intensity on the amplitude and reproducibility of the SEP responses on one participant. 15 different stimulation variants were explored, consisting of monophasic anodic electrical pulses in combinations of 3 pulse widths and 5 intensities. Four pulse intensities were defined relatively to the sensation threshold (1.5, 2, 2.5 3 times the sensation threshold) and the 5th intensity was set just below the pain threshold. The sensation threshold was defined as the level at which the subject was able to sense half of the 10 pulses given. The width of pulses tested was 100, 400 and 500 μs. This choice was made after reviewing the literature: 100 μs was the shortest pulse width suggested by a review study (Cruccu et al. 2008), 400 μs was the pulse width used in a recent study with similar analysis (Houzé et al. 2011) and 500 μs can be considered the mean width of electrical pulses across a variety of studies. Each of the 15 experimental conditions consisted of 500 trials divided in 5 experimental blocks, giving 75 blocks of 100 trials. The order of the total number of blocks (75) was randomized in order to minimize habituation effects.

A measure of reproducibility *r* was obtained by computing the spatio-temporal correlation coefficient between two averages of distinct random subsets of SEP responses (see “Statistics”).

Figure 6 shows the correlation coefficient, computed between two averaged non-overlapping datasets created with the bootstrapping method for the time window 25–65 ms after stimulus onset. Mean and standard deviation of the bootstrapped based coefficients are depicted for the different combinations of pulses and intensities. The correlation coefficient of the pulse width 400 µs turned out more stable over intensities than for the other pulse widths. Moreover, the amplitude of the peaks (see Table 4) for the pulse width of 400 μs and at a threshold of two times the sensation threshold was larger than for the other two pulses’ widths. We selected 400 μs as the pulse width for our experiment and as a stimulation threshold we selected the two times sensation threshold as it was not yet uncomfortable for the subject.

Figures 7, 8, 9, 10 show the GFP computed in μV^2^ (Brunet et al. 2011) for different fingers and for subjects # 1, 2, 3, 4, subject 5 and the grand average of SEP responses of the 5 subjects is presented in Fig. 2.

**Table 4:** Width of the electrical stimulation pulse test: report on SEP latency and amplitude

Pulse width (μs)	Times × sensory threshold	Intensity (mA)	Latency 30–60 ms	Amplitude 30 ms in μV
100	1.5 sensory	3	46	0.17
100	2 sensory	3.5	38	0.11
100	2.5 sensory	4	40	0.63
100	3 sensory	5.5	40	0.75
100	Below pain	7.5	38	0.32
400	1.5 sensory	1	*	*
400	2 sensory	1.5	42	0.5
400	2.5 sensory	2	43	0.74
400	3 sensory	2.5	37	0.22
400	Below pain	3	37	1.1
500	1.5 sensory	1	43	0.34
500	2 sensory	1.5	45	0.23
500	2.5 sensory	2	45	0.23
500	3 sensory	2.5	35	0.32
500	Below pain	3	38	0.50

The latency and amplitude of all the experimental conditions (15 pulse width-amplitude combinations) for the main peak in the global field power are presented and expressed in μV. The symbol * indicates a case in which we failed to identified the peak
